# Diagnosis and Treatment of Bone Disease in Multiple Myeloma: Spotlight on Spinal Involvement

**DOI:** 10.1155/2013/104546

**Published:** 2013-12-08

**Authors:** Patrizia Tosi

**Affiliations:** Hematology Unit, Department of Oncology and Hematology, Infermi Hospital, Viale Settembrini 2, 47100 Rimini, Italy

## Abstract

Bone disease is observed in almost 80% of newly diagnosed symptomatic multiple myeloma patients, and spine is the bone site that is more frequently affected by myeloma-induced osteoporosis, osteolyses, or compression fractures. In almost 20% of the cases, spinal cord compression may occur; diagnosis and treatment must be carried out rapidly in order to avoid a permanent sensitive or motor defect. Although whole body skeletal X-ray is considered mandatory for multiple myeloma staging, magnetic resonance imaging is presently considered the most appropriate diagnostic technique for the evaluation of vertebral alterations, as it allows to detect not only the exact morphology of the lesions, but also the pattern of bone marrow infiltration by the disease. Multiple treatment modalities can be used to manage multiple myeloma-related vertebral lesions. Surgery or radiotherapy is mainly employed in case of spinal cord compression, impending fractures, or intractable pain. Percutaneous vertebroplasty or balloon kyphoplasty can reduce local pain in a significant fraction of treated patients, without interfering with subsequent therapeutic programs. Systemic antimyeloma therapy with conventional chemotherapy or, more appropriately, with combinations of conventional chemotherapy and compounds acting on both neoplastic plasma cells and bone marrow microenvironment must be soon initiated in order to reduce bone resorption and, possibly, promote bone formation. Bisphosphonates should also be used in combination with antimyeloma therapy as they reduce bone resorption and prolong patients survival. A multidisciplinary approach is thus needed in order to properly manage spinal involvement in multiple myeloma.

## 1. Introduction

Multiple myeloma (MM) is a clonal B-cell disorder characterized by proliferation and accumulation of B-lymphocytes and plasma cells in the bone marrow and, more rarely, at extramedullary sites. Its annual incidence is 6/100000 in western countries, thus representing the second most common hematological malignancy after non-Hodgkin lymphomas [1]. Bone disease occurs in approximately 80% of patients with newly diagnosed MM, and in 70% of the cases bone pain is the first symptom to be reported at disease onset [2]. Pathological fractures, osteolyses, osteoporosis or, in general, skeletal-related events (SRE), that include also the need for radiotherapy or surgery to the bone, can severely impair patients quality of life and reduce survival [3]. Spine is the bone site that is most frequently affected by MM-related lesions [4]. Vertebral lesions can result in pain, permanent deformity, kyphosys, walking impairment, permanent disability, or paralysis. The aim of the present review is to describe the modality by which vertebral lesions occur in MM and to discuss the most appropriate therapeutic approaches.

## 2. Pathogenesis of MM-Related Bone Lesions

Normal bone homeostasis is maintained by a balanced and continuous remodeling process performed by the coordinated activity of osteoclasts and osteoblasts. Osteoclasts are macrophage derived cells that interact with bone surface with a highly specialized portion of their cell membrane called ruffled border and produce metalloproteinases and other proteolytic enzymes capable of degrading bone matrix [5]. Osteoblasts are mesenchymal derived cells that produce bone matrix and finally differentiate to osteocytes [6]. The mechanism that causes bone disease in multiple myeloma is based upon the fact that neoplastic plasma cells, either directly or indirectly through their interaction with bone marrow stromal cells, induce an alteration in the mechanisms of bone remodeling, as demonstrated by in vitro coculture experiments [7], so that bone resorption is promoted (increased osteoclast activity) and bone formation is inhibited (reduced osteoblast activity). It is well known that osteoclasts are recruited and undergo normal maturation through the interaction of their receptor RANK (receptor activator of nuclear factor *κ*B) with its ligand (RANK-L) produced by stromal cells, preosteoblasts, and activated T-lymphocytes [7]. The activity of RANK-L is balanced by the presence of its decoy receptor, osteoprotegerin (OPG) produced by stromal cells, and preosteoblasts [7, 8]. In MM, osteoclast activity is promoted by an increased production of RANK-L by stromal cells and preosteoblasts, a reduced production of OPG, and the upregulation of proosteoclastogenic cytokines such as Interleukin 1 (IL1)-alpha, macrophage-colony stimulating factor (M-CSF), and macrophage inflammatory protein (MIP)-1-alpha [7, 8]. This latter cytokine can activate monocytes, thus recruiting osteoclast progenitors and promoting their differentiation to mature osteoclasts [9]. Another recently identified proosteoclastogenic cytokine is activin A, a tumor-growth-factor- (TGF-) beta family member, that promotes osteoclast differentiation and inhibits osteoblast maturation [10]. The activity of osteoblasts is further reduced as malignant bone marrow plasma cells can express and secrete DKK-1, a soluble inhibitor of wnt signaling inhibitor that could potentially impair the maturation of osteoblasts [11]. Another mechanism that could contribute to impair osteoblastogenesis is the reduced production of RUNX-2-CBFA1, a transcription factor that plays a central role in promoting osteoblast maturation [12]. Osteoclasts can in turn stimulate plasma cell growth through an increased production of IL-6 [13, 14], thus contributing to the maintenance of the vicious circle.

### 2.1. Spinal Involvement in Multiple Myeloma

**Figure 1:**
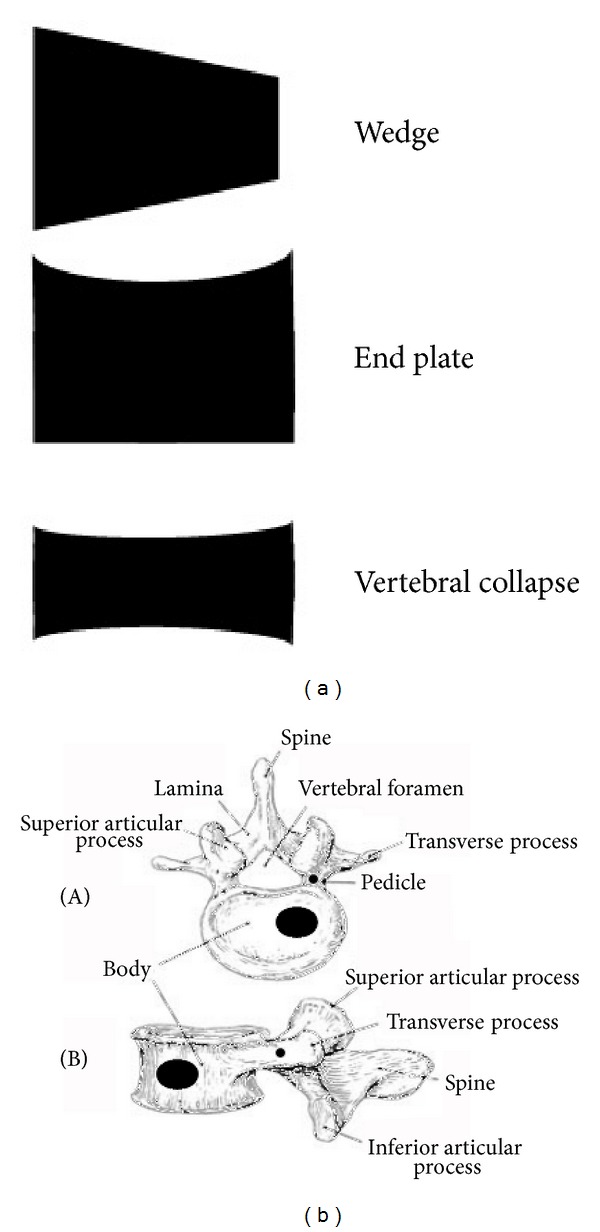
Shape of vertebral fractures (a) and localization of osteolyses within the vertebral body (b).

**Figure 2:**
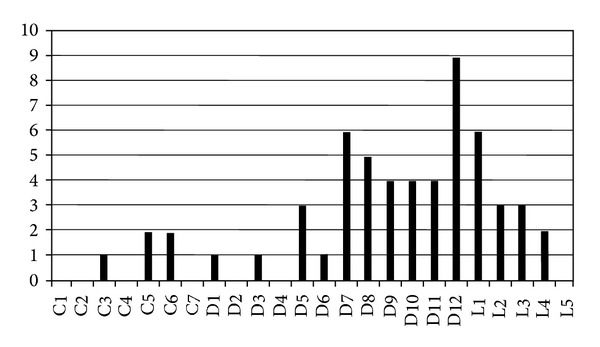
Frequency of vertebral fractures at different vertebral levels. Analysis of 57 newly diagnosed MM patients followed at the Hematology Unit, Rimini Hospital, Italy.

**Figure 3:**
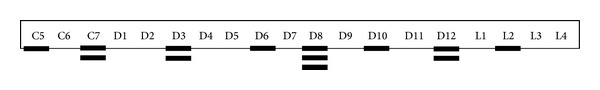
Occurrence of vertebral masses at different vertebral levels. Analysis of 13 newly diagnosed MM patients followed at the Hematology Unit, Rimini Hospital, Italy.

As it has been described for solid tumor metastatic to the bone, vertebral lesions are frequently observed in MM patients. It can be estimated that over 60% of bone lesions occurring in MM patients involve the spine, as compared with 90% in metastatic prostate cancer, 75% in breast cancer, and 45% in lung cancer [15]. This could be attributed to the fact that vertebral bodies contain a high amount of hematopoietic bone marrow, so that a large surface of the hematopoietic niche is adjacent to oteoblasts, osteoclasts, or other stromal cells involved in bone remodeling. The existance of a close relationship between osteoblastogenesis and myelopoiesis has been demonstrated in different early studies mainly conducted in animal models; in particular, a higher amount of hematopoietic stem cells can be found in close proximity to stimulated osteoblastogenesis [16] and, conversely, when osteoblasts growth and maturation are impaired, hematopoietic cells show growth defect [17]. Furthermore, as described above, a reciprocal stimulation has been described between neoplastic plasma cells and osteoclasts [13, 14]. Vertebral involvement in MM can appear as generalized osteoporosis or as osteolyses that are located in vertebral bodies or, more rarely, in transverse processes, spinous processes, or pedicles (Figure 1). Vertebral fractures appear as endplate alterations, wedge deformities, or vertebral collapses (Figure 1). Over 80% of the vertebral fractures occur in D6-L4 region of the spine, and 50% of them can be found in the D11-L1 region [4]. This is similar to what is observed in patients with benign osteoporosis, and it is probably due to the contribution of biomechanical factors acting at the dorsal-lumbar transition (Figure 2). All the same, the shape of the lesions can vary depending on the site of the spine that is involved; vertebral collapses are found mainly in the dorsal region, endplate lesions in the lumbar region, and wedge lesions in the dorsal-lumbar transition [4]. Cytogenetic evaluation of the plasma cells infiltrating vertebral lesions and those collected with random biopsies showed the same chromosomal alterations or a more aggressive genotype in focal vertebral lesions [18]. More rarely, MM can manifest with masses arising from vertebral bodies; they can expand either in epidural or in paraspinal regions leading to spinal cord or dorsal nerve roots compression [19]. At variance to what has been reported for fractures, vertebral masses can be found everywhere in the spine, even in cervical vertebrae, which are more rarely involved by fractures [4] (Figure 3).

The spine is also the site where bone solitary plasmacytomas are more frequently observed; the average incidence is 50% as compared to 12% for the pelvis and 9% for the ribs [20]. Dorsal and lumbar spine are more frequently affected, while cervical spine is seldom involved [21].

### 2.2. Monoclonal Gammopathy of Uncertain Significance (MGUS) and Vertebral Lesions

Subjects with MGUS have, by definition, a serum monoclonal protein <3 g/dL, less than 10% clonal plasma cells in the bone marrow and absence of end-organ damage, such as hypercalcemia, anemia and, more specifically, bone lesions that can be attributed to the plasma cell proliferative disorder [22]. Several lines of evidence, however, pointed out that bone mineral density can be impaired in these patients [23]. A retrospective population-based study has revealed that MGUS can be detected in a high percentage of patients with a confirmed diagnosis of osteoporosis [24]. Another study performed in 65 women with MGUS showed that lumbar spine bone mineral density and serum RANK-L/OPG ratio were related to the duration of MGUS [25]. It could be argued that the incidence of both MGUS and osteoporosis increases with age, so that it can be difficult to ascertain whether MGUS can really cause a disruption of bone metabolism. In a subsequent report it has been demonstrated that the risk of vertebral fractures was increased in MGUS patients as compared to matched general population, and this has not been observed for appendicular fractures [26]. Although the contribution of other risk factors such as older age and steroid use cannot be overlooked, this finding is unexplained by the present knowledge on the biology of MGUS and poses the question of using bone-protecting agents earlier in the course of the disease.

### 2.3. Spinal Cord Compression

Epidural spinal cord compression (SCC) occurs in up to 20% of patients with MM at various disease stages [27]. The pathogenetic mechanisms are induced by displacement and compression of the spinal cord, and this can be caused by either epidural invasion by neoplastic tissue arising from a vertebral mass, as described above, or by osseous fragments protruding from a fractured vertebral body. Pain is the first and more common presenting symptom [28, 29]. It is generally a mechanical pain caused by periosteal infiltration of the vertebrae, it becomes more intense in case of cough or labor, and it is further exacerbated when exerting pressure on the spinous processes. Radicular pain can also be present [28, 29]; this can be caused by nerve-root compression and it is perceived according to the dermatomal distribution of the nerve root. Motor dysfunction is the second more frequent symptom of SCC. Patients complain about weakness of lower limbs, in particular when walking or going up the stairs. Sensory symptoms such as paresthesias, tingling, or numbness can occur simultaneously or after motor dysfunction; they usually precede autonomic-sphinteric symptoms that are usually represented by bladder dysfunction [28, 29]. Prompt recognition of these symptoms and subsequent intervention is mandatory as the picture invariably proceeds to paralysis that is frequently irreversible [30]. The gold standard diagnostic procedure to evaluate SCC is spinal magnetic resonance imaging (MRI), which allows a clear identification of bone lesions, tumor masses, and neural alterations [31]. Regarding therapeutic approaches, decompressive laminectomy was frequently performed in the past but its use is now abandoned due to the residual instability of the vertebral column, to the possible delay in the beginning of antimyeloma therapy after surgery and, above all, to the sensitivity of neoplastic cells to steroids and radiotherapy, that now represent the mainstay of the treatment of SCC [28, 32]. High-dose steroids, such as Dexamethasone at doses of 40–60 mg/day for 4–6 days must be soon initiated upon recognition of SCC, aiming at obtaining both a plasmacytolitic and an antioedema effect. Radiotherapy, either 30 Gy in 10 fractions or shorter courses [32], must be also administered early, as an optimal and long-lasting local control of the disease can be achieved.

## 3. Diagnosis

### 3.1. Imaging

Many studies have been conducted in order to assess the best strategy to identify bone lesions in MM, even though few of them were specifically addressed at evaluating the axial skeleton. Whole body skeletal X ray (WBXR) has long been considered the standard for the detection of MM-related bone disease, even though lesions become evident when over 20% of trabecular bone is lost, thus leading to underestimation of initial lesions [33]. Despite that, WBXR is still considered mandatory by major MM study groups in order to make a correct diagnosis of symptomatic disease [34, 35] and is invariably included in the diagnostic workups of clinical trials. Another pitfall of this technique is represented by its scarce reliability for patients follow-up, as improvement of bone lesions can be rarely assessed [36]. Computed tomography (CT) allows a higher detection rate of bone lesions due to its high sensitivity for the evaluation of alterations in bone mineralization [34, 35, 37]. Its use, however, is frequently limited to a definite vertebral level, especially in preparation to fine needle biopsy of a suspected lesion. This is due to two major drawbacks of this technique: first of all no functional information can be achieved as the evaluation of disease activity at the site of the lesions is not feasible, second, the radiation dose, which is higher than that delivered after WBXR [37]. Recently, however, low-dose whole body CT has been introduced in the clinical practice [37, 38], and the International Myeloma Working Group (IMWG) has suggested the potential of this technique to replace WBXR [35]. MRI is the most sensitive and specific imaging technique to evaluate spinal lesions [39], as it allows morphological detection of vertebral compression fractures together with spatial evaluation of neural damage or paraspinal masses. The most interesting feature, however, is the possibility to evaluate the characteristics of bone marrow infiltration by the disease. MM-related vertebral focal lesions present with a diffusely reduced signal in T1-weighted images and enhanced in T2-weighted images; bone marrow infiltration can thus be defined as “focal” when a clear number of lesions can be identified in the context of a normal background; “diffuse” when all the bone marrow shows an altered signal, and “mixed” when both focal lesions and diffuse alteration are present [4, 40]. MRI presents several advantages over the other imaging techniques for the diagnosis and monitoring of spinal alterations in MM related bone disease. First of all the possibility of detecting initial lesions in either symptomatic patients with negative WBXR or in asymptomatic patients with full blown MM and, again, a negative WBXR. Furthermore, with MRI it is possible to differentiate a pathological fracture from one caused by benign osteoporosis, and this is extremely useful when treating elderly female patients with preexisting vertebral lesions [4, 39]. MRI is also useful to monitor the efficacy of the treatment, as bone marrow infiltration can normalize in case of response [39, 41, 42]. Finally, bone lesions detected at MRI seem to possess a prognostic role in different stages of the disease. Patients in stage I MM and focal lesions detected at MRI have a shorter time to progression as compared to patients with a negative MRI [43]. The same finding was demonstrated also in subjects with asymptomatic disease [44]. In patients with symptomatic disease, a poorer prognosis was observed in case more than 7 focal lesions were detected at spinal MRI [45] or when a diffuse pattern of bone involvement was observed [42]. Although these findings indicate that spinal MRI possesses a diagnostic and prognostic role in MM, its routine use is not recommended in clinical practice, and the IMWG suggests its use only in patients with vertebral symptoms and a negative WBXR [35]. Several studies have recently addressed the issue of fluorodeoxyglucose- (FDG-) positron emitting tomography (PET) in MM [46–49]. The major limitation of this technique is the low proliferative activity of neoplastic plasma cells, thus leading to a modest glucose utilization that results in a reduced standardized uptake value (SUV) as compared, for instance, to lymphomas [46]. In order to potentiate its specificity, PET is now used in conjunction with CT (PET-CT), thus allowing a better spatial definition of the lesions. This technique has demonstrated to be less sensitive than MRI in detecting small vertebral alterations [48]; however it seems to possess a definite prognostic role in newly diagnosed symptomatic MM, as patients showing >3 focal lesions have a shorter survival rate [47]. Furthermore, disappearance of FDG-PET positive lesions after high-dose therapy and autologous stem cell transplant predicts a more prolonged disease control [49].

## 4. Treatment 

Treatment of MM-related spinal lesions is based upon a multidisciplinary approach, as both medical, surgical, and minimally invasive techniques are employed. Medical therapy is aimed at treating bone disease in general, while other approaches are mostly targeted to the specific vertebral lesions.

### 4.1. Antimyeloma Therapy

As disruption of bone metabolism in MM is caused by the interaction of neoplastic plasma cells with bone marrow stroma, it can be hypothesized that antineoplastic therapy, when effective, could restore a normal bone remodeling. This cannot be the cause when high-doses dexamethasone are used, as steroids are known to suppress osteoblastogenesis, to induce osteoblast apoptosis, and to downregulate OPG, thus allowing the interaction of RANK-L with RANK, which is subsequently activated and promotes the proliferation of preosteoclasts and the activation of mature osteoclasts [50]. A further bone loss is then expected in patients treated with high-dose dexamethasone, and this should be counteracted with the concomitant use of bone-protecting agents such as bisphosphonates. No studies have been reported concerning the effects of conventional chemotherapy alone at standard doses on bone metabolism. On the other hand, it has been reported that high-dose myeloablative therapy and autologous stem cell transplant can reduce osteoclast activity, as demonstrated by a progressive reduction of markers of bone resorption through the various phases of the treatment program [51].

Drug combinations targeting both myeloma cells and bone marrow microenvironment could be potentially useful in inducing disease response and halting bone resorption [52]. In this setting, thalidomide and lenalidomide represent a new treatment paradigm because of their alternative mechanism of action that includes disruption of the interaction between plasma cells and bone marrow stromal cells, inhibition of cytokine secretion, antiangiogenic activity, and immunomodulatory effects [53]. These drugs interact with bone marrow stromal cells and inhibit the production of cytokines that are known to be directly involved in osteoclastogenesis, including IL-6, IL-12, vascular endothelial growth factor (VEGF), and tumor necrosis factor (TNF)-alpha [52], so that an important role in inhibiting osteoclast recruitment, maturation, and activity can be postulated. Upon treatment with a combination of thalidomide and dexamethasone, a reduction of serum and urine markers of bone resorption has been demonstrated in newly diagnosed patients [54], especially in cases showing a greater tumor response; in relapsed and refractory patients a progressive decrease of the sRANKL/OPG ratio was also observed during the course of the treatment with thalidomide plus dexamethasone [55]. Similar results in terms of decrease of sRANK-L/OPG were demonstrated in patients treated with lenalidomide-dexamethasone [56], together with a reduction of preosteoclast growth upon in vitro culture of freshly isolated bone marrow mononuclear cells from treated patient. A possible mechanism of inhibition of osteoclastogenesis has also been proposed for the third generation thalidomide analog pomalidomide. In vitro culture of bone marrow mononuclear cells in the presence of the drug showed colony-forming unit granulocyte-macrophage (CFU-GM) growth inhibition. Molecular analysis performed in the same cells showed a reduced expression of PU-1, a transcription factor that promotes macrophage maturation [57], so that an inhibition of the preosteoclastic compartment has been suggested [58]. Bortezomib, the first in class proteasome inhibitor that is now considered one of the most important drugs in the treatment of MM, both at diagnosis and in relapsed-refractory patients [59, 60], seems to possess a peculiar activity on bone disease. The drug, as other compounds acting on both neoplastic plasma cells and bone marrow stroma, is able to reduce bone resorption markers in vivo, in relapsed refractory patients [61]. In variance to what has been observed with other novel compounds, several studies suggested a possible bortezomib-induced promotion of bone formation. It was initially reported in two studies that bone alkaline-phosphatase was increased in patients both responsive and resistant to bortezomib-dexamethasone combination [62, 63], and subsequent studies conducted in mice demonstrated a significant increase in bone mineral density of treated animals [64]. This osteoblast-stimulating activity was further confirmed in larger studies conducted in patients treated with bortezomib [65], even though the mechanism of these effects has not been clarified yet; differentiation from mesenchymal cells, probably promoted by an increase of RUNX-CBFA1 transcription factor [66], and upregulaton of vitamin D3 signalling [67] seem to be involved.

### 4.2. Bisphosphonates

Bisphosphonates (BPs) are at present the only compounds routinely used in the clinic that possess a specific inhibitory activity on osteoclast-mediated bone resorption [68, 69]. These drugs are pirophosphate analogues in which the central oxygen bridge has been replaced by a carbon that is linked to different side chains. In first generation bisphosphonates, like clodronate, small radicals are linked to the carbon, while in second generation bisphosphonates nitrogen containing moieties, either simple (as in pamidronate) or more complex (as in zoledronic acid) are found. Bisphosphonates bind avidly to the bone mineral matrix and therefore accumulate in bone at sites of active bone metabolism [68, 69]. First generation BPs enter osteoclasts and inhibit ATP-mediated intracellular processes. Nitrogen-containing bisphosphonates (N-BPs) have a different mechanism of action as they exert their cellular effects via inhibition of protein prenylation [70]. In vitro studies have shown that N-BPs inhibit the activity of farnesyl diphosphonate (FPP) synthase, a key enzyme in the mevalonate pathway, thus disrupting prenylation of small intracellular guanine triphosphatases, which are essential for cell function and survival. As a result, N-BPs inhibit osteoclast activity by interfering with intracellular processes such as organization of the cytoskeleton, membrane trafficking, and formation of the ruffled border [70, 71]. Among the N-BPs tested, zoledronic acid was the most potent inhibitor of FPP synthase, producing near-complete inhibition of enzyme activity at a concentration of 0.1 *μ*M (Table 1). N-BPs inhibit osteoclastogenesis and recruitment of osteoclast progenitors to the bone and induce osteoclasts apoptosis, probably by activation of caspases [71].

BPs have been introduced in the clinic for the treatment of MM-related bone disease in the early eighties, from then on their use has progressively increased and they are now considered an essential component of the whole treatment approach of MM patients. Among first generation BPs, oral clodronate has demonstrated to significantly reduce skeletal-related events (SRE) in newly diagnosed symptomatic patients as compared to placebo [72]. Intravenous pamidronate 90 mg/month has as well demonstrated to be more effective than placebo in a similar subset of patients, and the data were confirmed also in relapsed and refractory patients [73, 74]. A more recent study conducted in over 500 patients with newly diagnosed MM showed that pamidronate 30 mg/month was as effective as 90 mg/month in terms of time to SRE or SRE-free survival [75]. A large double blind randomized trial has demonstrated that zoledronic acid was at least noninferior to pamidronate in reducing the incidence of SRE, in delaying the time to first SRE, and in reducing bone pain in newly diagnosed MM patients treated with conventional chemotherapy [76]; a significant delay in time to first radiation treatment was shown in the zoledronic acid arm [77]. A large randomized trial comparing clodronate and pamidronate in addition to first-line therapy was recently conducted by the Medical Research Council (MRC) [78]. This study gave interesting insights on the role of BPs in MM as it evaluated the different settings of application of the drugs in newly diagnosed patients. Zoledronic acid has demonstrated to be superior to clodronate in delaying SRE in all the patient subsets, specifically in transplant candidates and in nontransplant candidates, in subjects undergoing thalidomide maintenance or not, and, most importantly, in patients presenting with or without bone lesions at diagnosis [79]. This latter finding underlines the importance of adding BPs when setting up MM therapy in all newly diagnosed symptomatic patients. Several studies conducted in vitro and in vivo, in preclinical models, demonstrated that BPs, and in particular the more potent N-BPs, also have antitumor activity. Specifically BPs can inhibit proliferation and induce apoptosis in vitro in different human MM cell lines or freshly isolated plasma cells from MM patients [80–82]. Inhibition of tumor cell adhesion, invasion of the extracellular bone matrix, and angiogenesis have been also described [81, 82]. N-BPs appear also to possess a variety of immunomodulatory effects that may contribute to their antitumor activity. In animal studies, N-BPs were shown to enhance production of inflammatory cytokines by antigen-presenting cells and to overcome tolerance to tumor antigens. In addition, N-BPs stimulate the proliferation of a specific gamma/delta T-cell subset [83], and these T cells exhibited cytotoxic activity against a number of tumor cell lines. It has been difficult to reproduce these interesting data in the clinic. A subanalysis of the trial comparing clodronate versus placebo demonstrated a prolonged survival in patients with no vertebral fractures at diagnosis and subsquently treated with clodronate [72]. In the long-term followup of a randomized, placebo-controlled trial of pamidronate (90 mg) for treatment of advanced multiple myeloma, subset analysis revealed a trend toward a survival advantage in a subgroup of patients who were receiving second-line chemotherapy or greater [74]. The MRC trial mentioned above [78, 79] demonstrated that the combination of zoledronic acid to first-line therapy reduced the risk of death and prolonged survival in treated patients as compared to clodronate. A recent meta-analysis of the Cochrane group [84] showed that zoledronic acid was the only BPs associated with superior OS as compared to placebo, but not in comparison with other BPs.

Although BPs are not recommended in patients with asymptomatic MM or MGUS [85], several studies were performed in order to assess whether these categories of patients could benefit from an early medical intervention aimed at preventing bone disease. In patients with asymptomatic MM, two trials were conducted in order to assess whether BPs could reduce the time to first SRE or to progression to symptomatc disease. Both intravenous pamidronate [86] and zoledronic acid [87], administered monthly for one year, failed to determine any advantage in time to progression; conversely, time to first SRE was significantly extended. As reported above, MGUS subjects show an increased risk of vertebral fractures as compared to general population; two studies were carried out using intravenous zoledronic acid (three 4 mg doses at 4–6 months intervals) [88] or oral alendronate (70 mg/week) [89]; in both cases an increase in bone mineral density at lumbar spine was observed.

BPs are generally well-tolerated; gastrointestinal discomfort and acute-phase reactions are the most frequent side effects observed in patients treated with oral or intravenous BPs, respectively [84, 85]. Kidney damage, though less common, should be carefully avoided by close monitoring of renal function, reducing the dose or the infusion rate of the compounds [74–76]. Osteonecrosis of the jaws (ONJ), a non-healing area of exposed bone in the oral cavity, is a serious complication that was recognized in the late nineties as to be related to BPs treatment [90]. Retrospective studies reported a 4–9% incidence of ONJ; this was more common in patients treated with zoledronic acid and had a direct relationship with treatment duration [91–93]. Major risk factors were poor oral hygiene, invasive dental procedures, and local infections [91–93]. Implementation of dental prophylactic mesaures prior to and during BPs therapy has significantly reduced the incidence of this complication [94, 95].

### 4.3. Novel Drugs Acting Specifically on Bone Disease

Denosumab is a fully human monoclonal antibody that targets RANK-L and has been introduced in the treatment of metastatic solid tumors and MM-related bone disease [96]. This drug is able to inhibit osteoclastogenesis in vivo, as demonstrated by the rapid and sustained decrease in markers of bone resorption [97]. A larger randomized trial aimed at comparing denosumab with zoledronic acid conducted in patients with MM and solid tumors excluding breast and prostate cancer demonstrated that denosumab was noninferior to zoledronic acid in delaying time to first skeletal-related event, while the incidence of osteonecroisis of the jaw was similar [98]. Two other promising compounds are presently under investigation. As described above, activin A is a TGF-beta family member known to stimulate osteoclastogenesis and to inhibit osteoblast maturation [10]. Specifically, activin A is produced mainly by bone marrow stromal cells and osteoclasts and its serum levels are increased in the bone marrow of patients with MM and bone osteolytic lesions [10]. Recently, a human antiactivin A monoclonal antibody was made available and tested in a phase II clinical trial [99]. Early data show that treated patients had an improvement in hemoglobin level and increase in bone formation markers [100]. Another promising drug is an anti-DKK1 monoclonal antibody that, in vitro, seems to reverse the inhibitory effect of MM cells on osteoblastogenesis [101].

### 4.4. Radiotherapy

External beam radiation therapy represents the treatment of choice for solitary plasmacytoma of the bone [102, 103]. In MM, radiation to the spine is usually employed in patients with uncontrolled pain or in case of impending vertebral fracture or spinal cord compression. Early studies that were conducted in small patient cohorts demonstrated that pain relief, quality of life, and motor function were improved in a sizeable proportion of treated cases [104, 105]. No difference in general efficacy and in the extent and rapidity of pain relief has been observed using a fractionated two-week course of 30 Gy or a single fraction of 8–10 Gy [106]. Radiation field should be large enough to compensate patient motion, but it should also be as limited as possible in order to preserve marrow function in patients concomitantly treated with systemic cytoreductive therapy. This is especially true for patients who are candidates to autologous stem cell transplant as peripheral blood stem cell collection can be severely impaired when radiotherapy is applied in large fields [107].

### 4.5. Surgery

Surgical management of MM-related vertebral lesions is seldom carried out due to the chemoradiosensitivity of the disease and to the morbidity potentially associated with the procedure, which can result in a delay in the initiation of systemic cytoreductive therapy. The only indications for surgical intervention are unstable fractures, SCC, especially when it is caused by a radioresistent mass, or by bone fragments protruding from a vertebral fracture [108]. In the past, decompressive laminectomy using a posterior surgical approach was the treatment of choice, but nowadays it is rarely employed as it can cause destabilization of the spine and consequently increase in pain and neurological alterations [109]. Vertebral stabilization, which is generally carried out by means of appropriate titanium cages, is now considered mandatory after surgery [110]. Furthermore, depending on the vertebral levels that are involved by the lesions, an anterior or transpeduncolar surgical approach can be used, thus resulting in a better patients outcome [111].

### 4.6. Vertebral Augmentation

Vertebral augmentation techniques, namely, vertebroplasy and kyphoplasty, are carried out by fibroscopic percutaneous injection of polymethylmetacrilate (PMMA) into the fractured vertebrae, in order to relieve bone pain. Percutaneous vertebroplasty consists of the direct injection of PMMA into the damaged vertebral body, while kyphoplasty is performed by inserting on the vertebral body and inflatable balloon that is subsequently filled with PMMA [112]. Vertebroplasty was introduced in Europe almost thirty years ago, and it was initially proposed as a treatment for vertebral fractures due to benign osteoporosis [113]; after demonstration of its efficacy in achieving pain relief [113, 114], it was extensively used in the treatment of vertebral lesions caused by solid tumor metastatic to the bone and MM [115–117]. Different studies reported that over 80% of the patients can percieve a significant improvement in rest or activity pain [115–117]. Positive results were maintained after medium or long-term observation [117]. The major complication of vertebroplasty is cement leakage from the damaged vertebrae, that indeed is a rare event but it can lead to further different serious complications, including intractable vertebral pain [115–117] and pulmonary embolism. Balloon kyphoplasty was introduced more recently in the clinical practice; in addition vertebroplasty has the advantage of a lower probability of cement leakage and a better restoration of vertebral height [118], while pain response is similar to what can be obtained with vertebroplasty, thus approaching 90% [118, 119]. The main disadvantages are represented by the higher costs and by the complexity of the whole procedure.

Finally, for both the procedures several recommendations should be kept in mind. Vertebral augmentation should be performed as early as possible, in order to improve the vertebral strength and to avoid further stress fracture due to alteration in the mechanics of spine; for the same reason, treatment of more than 3 vertebral levels at a time is not recommended as a rapid change in the shape of the spine may occur, thus increasing the risk of stress fractures. In case radiotherapy is planned, it should be performed after vertebroplasty or kyphoplasty. None of the two vertebral augmentation techniques should be carried out in case of retropulsed posterior wall, vertebral instability, and SCC.

## 5. Concluding Remarks

In recent years the outcome of MM patients has significantly improved due to the widespread use of autologous stem cell transplantation [120, 121] and novel therapies targeting both the myeloma clone and its microenvironment [122]. Despite that, control of bone disease does still represent a therapeutic challenge in these patients. Over two-thirds of the patients with MM present, at some time during their disease course, osteopenia, osteoporosis, or pathological fractures, that in over 60% of the cases involve the spine at its various levels [4], and this can result in significant patients morbidity, ranging from disabling pain to SCC. Diagnosis of vertebral lesions must be rapid in order to avoid further complications, and the site and the morphology of the lesion should be identified as precisely as possible. The management of vertebral lesions should take into account the therapeutic program that the patient will subsequently receive. Surgical approaches can be employed in case of SCC, but they are not considered a feasible choice when a less severe vertebral involvement is present, as the time lag necessary to recover after the intervention results in a delay in the initiation of cytoreductive therapy. All the same, except in case a rapid debulking of a vertebral mass is required, radiotherapy is generally not applied in large fields, due to the toxic effects exerted on bone marrow stem cells, leading to severe impairment in PBSC mobilization and collection [107]. Percutaneous vertebroplasty or balloon kyphoplasty lead to rapid and durable pain control without interfering with systemic treatment. Early establishment of medical therapy is essential in controlling MM-related bone disease; this is accomplished with bisphosphonates, which promote inhibition of osteoclasts maturation and function [85], and, indirectly, with antimyeloma therapy, which reduces the stimulation of osteoclastogenesis exerted by neoplastic plasma cells [51–67]. Induction therapy with novel drug combinations targeting both myeloma cells and stromal cells, such as thalidomide or lenalidomide has demonstrated to be useful in inducing disease response and blocking bone resorption [54–58]. Bortezomib, on the other hand, has also proven to be effective in promoting bone formation by stimulation of osteoblast maturation [62–67]. It can be thus concluded that a multidisciplinary approach is required for the diagnosis and treatment of vertebral lesions in MM, and cooperation between experts (hematologists, radiologists, orthopedists, radiotherapists, physiatrists, and neurologists) is mandatory for an optimal management of the patients.

## Figures and Tables

**Table 1:** Relative potency of different bisphosphonates.

	Relative potency	
	In vitro	In vivo
Etidronate	1	1
Clodronate	8	10
Pamidronate	550	100
Alendronate	700	700
Ibandronate	5000	4000
Zoledronic acid	10000	10000
